# Role of Matrix Gla Protein in the Complex Network of Coronary Artery Disease: A Comprehensive Review

**DOI:** 10.3390/life11080737

**Published:** 2021-07-24

**Authors:** Marko Kumric, Josip A. Borovac, Tina Ticinovic Kurir, Dinko Martinovic, Ivan Frka Separovic, Ljupka Baric, Josko Bozic

**Affiliations:** 1 Department of Pathophysiology, University of Split School of Medicine, 21000 Split, Croatia; marko.kumric@mefst.hr (M.K.); jborovac@mefst.hr (J.A.B.); tticinov@mefst.hr (T.T.K.); dinko.marinovic@mefst.hr (D.M.); if91603@mefst.hr (I.F.S.); 2 Department of Cardiology, University Hospital of Split, 21000 Split, Croatia; 3 Department of Endocrinology, Diabetes and Metabolic Diseases, University Hospital of Split, 21000 Split, Croatia; 4 Institute of Emergency Medicine of Split-Dalmatia County (ZHM SDZ), Spinčićeva 1, 21000 Split, Croatia; ljupka.baric@zhmsdz.hr

**Keywords:** matrix Gla protein, coronary artery disease, vascular calcification, vitamin K

## Abstract

Coronary artery disease (CAD) is widely recognized as one of the most important clinical entities. In recent years, a large body of accumulated data suggest that coronary artery calcification, a process highly prevalent in patients with CAD, occurs via well-organized biologic processes, rather than passively, as previously regarded. Matrix Gla protein (MGP), a vitamin K-dependent protein, emerged as an important inhibitor of both intimal and medial vascular calcification. The functionality of MGP hinges on two post-translational modifications: phosphorylation and carboxylation. Depending on the above-noted modifications, various species of MGP may exist in circulation, each with their respective level of functionality. Emerging data suggest that dysfunctional species of MGP, markedly, dephosphorylated-uncarboxylated MGP, might find its application as biomarkers of microvascular health, and assist in clinical decision making with regard to initiation of vitamin K supplementation. Hence, in this review we summarized the current knowledge with respect to the role of MGP in the complex network of vascular calcification with concurrent inferences to CAD. In addition, we discussed the effects of warfarin use on MGP functionality, with concomitant implications to coronary plaque stability.

## 1. Introduction

Despite the fact that coronary artery disease (CAD) has been widely recognized as one of the most important clinical entities, certain aspects of the pathophysiological processes underlying this disease have not been completely elucidated [1]. One of those aspects is coronary artery calcification (CAC), the process of mineral deposition in the coronary vasculature previously regarded as passive and benign [2]. Nonetheless, in recent years, a large body of accumulated data suggests that CAC occurs via well-organized biologic processes, including an imbalance between osteochondrogenic signaling and anti-calcification events [3]. In line with this, it seems that CAC is highly prevalent in patients with CAD and that it is associated with the occurrence of major adverse cardiovascular events (MACEs) [4]. An important relation in this setting is between CAC and vascular stiffness. Namely, a firm and reciprocal correlation has been established between these two entities on both the preclinical and clinical level [5]. As vascular stiffness is a consequence of vascular tree damage caused by multiple CV risk factors, and it can thus be used as proxy for CV mortality prediction, vascular stiffness should be highlighted as much as hypertension in the future clinical perspectives of CAD management [5,6].

There are multiple mechanisms that mediate vascular calcification. One of the most discussed mechanisms in this setting is the failure of anti-calcification processes, either owing to the loss of or deficiency in the constitutively expressed mineralization inhibitors, such as matrix Gla protein (MGP), osteoprotegerin, osteopontin, and many others [3]. MGP, a small vitamin K-dependent protein, emerged as a potent vascular calcification inhibitor, and recent data suggest multiple implications of this protein in CAD development [7]. Hence, in this review we aimed to summarize the current knowledge with respect to role of MGP in the complex network of vascular calcification with concurrent inferences to CAD.

## 2. Pathophysiology of Vascular Calcification, Arterial Stiffness and Their Interrelation

In the traditional classification system, vascular calcification is divided into two distinct groups, based on the position of the mineral deposits [3]. Vascular calcification of the vessel wall can occur in either the intimal or medial layer of a blood vessel.

Intimal calcification is initiated by formation of microcalcifications—small (<5 µm) calcium deposits that accumulate as a result of necrotic or apoptotic cell death within the lipid core of atherosclerotic plaques [8]. This process is considered to arise from either apoptotic SMCs or exosomes released by SMCs near the internal elastic lamina. Rather interestingly, this process coincides with enhanced expression of the uncarboxylated form of MGP yet precedes the changes in the intimal content of the calcification-regulation proteins, such as osteocalcin and bone morphogenic protein-2 (BMP-2) [9]. Consequently, an increase in nucleation sites facilitates the precipitation of calcium salts at the microscopic level. Microcalcifications are very important in the setting of acute coronary events as they are commonly seen in high-risk (“vulnerable”) plaques. The pathophysiologic background to this observation lies in the fact that microcalcifications accumulate in “vulnerable” plaque, representing a calcific healing response similar to that occurring in tuberculosis, which attempts to reduce and wall off the inflamed necrotic environment, thereby reducing the risk of plaque rupture. However, in the early phases of microcalcification, this process could be detrimental, as data suggests that microcalcification might itself increase the propensity to rupture, acting as a focal point that intensifies mechanical stress on the atheroma cap [10]. If the plaque, however, ruptures, macrocalcifications (>5 µm) form on the site of the thrombotic occlusion, representing an important step in remodeling of the lesion [11].

The clinical importance of intimal calcification in the setting of CAD lies in the fact that calcification of atherosclerotic plaque represents a risk factor for plaque rupture [12,13]. However, it is noteworthy that this relation is controversial, as even though the amount of coronary calcification seems to have predictive value for CV events in various populations, the actual impact of calcification on plaque “stability” remains elusive [14,15]. In clinical terms, the visualized presence of calcium deposits within coronary vessels, quantified by the CAC score, showed a robust correlation with CAD [16,17,18,19]. A CAC score of 0 has been consistently associated with a very low risk of adverse CV events and low mortality, whereas very high CAC scores strongly indicate substantial CV risks and advanced plaque burden, as they are associated with increased risks of all causes of mortality, extensive coronary plaque burden, adverse CV events, and even cancer [20,21]. A strong link between calcium deposition and coronary plaque morphology and burden was also demonstrated in a seminal histopathologic study by Sangiorgi et al. [22]. The authors showed that coronary calcium quantification highly correlated with atherosclerotic plaque area within coronary vessels, but not with respect to lumen stenosis, thus showing that CAC quantity is a reliable correlate of atherosclerotic plaque presence and severity, rather than luminal narrowing. These findings rebounded in clinical practice as large CV societies nowadays endorse CAC screening as a highly distinct marker of coronary atherosclerosis and use it to guide the intensity of statin regimens among individuals with subclinical CV disease or those with intermediate risk for atherosclerotic CV disease [23,24]. Taken together, the important role of calcification in CAD development has been unequivocally proven from the basic aspects of anatomy and histopathology, as well as from a clinical standpoint.

Medial calcification, on the other hand, is a process most commonly seen in chronic kidney disease (CKD), but also with diabetes mellitus, hypertension, aging, and osteoporosis [25,26]. Rather interestingly, by affecting vascular stiffness, medial calcification can increase the incidence of CV complications even in the absence of vascular lumen stenosis [27,28,29]. The media of the blood vessel wall has two main components: SMCs and elastin-rich extracellular matrix (ECM). A critical process that enables the calcification of the media is differentiation of SMCs into osteoblast-like cells, a process that somewhat resembles bone formation. In fact, components implicated in this process (BMP-2, Msh Homeobox 2, and alkaline phosphatase (ALP)) are the same components that participate in bone remodeling [30]. The initiation of the above-noted process is enabled by reduction of calcification inhibitors, an increase in oxidative or endoplasmic reticulum (ER) stress, impaired SMC signaling, apoptosis, and disorder of the calcium-phosphate homeostasis which arises for hormonal dysregulation [31,32]. These processes warrant calcium depositions via production of matrix vesicles by SMCs [31]. Notable inducers and inhibitors of vascular calcification are listed in Table 1.

A firm and reciprocal correlation has been established between vascular stiffness and calcification in both basic and clinical studies [5,54,55,56]. The principal consequences of vascular stiffness increment are left-ventricular overload and impairment of coronary perfusion pressure, which naturally occurs during diastolic filling and increased transmission of pulsatile energy towards low-resistance organs, such as the kidneys and brain [57]. ECM stiffness, arising as a result of ECM calcification, leads to a decrease in production of nitric oxide (NO), the main culprit of the vascular stiffness, guiding to transdifferentiation of SMCs into the hypercontractile phenotype and stimulating endothelial cells to endothelial-to-mesenchymal transition (EndoMT) [58]. The newly formed mesenchymal stem cells then further differentiate into osteochondrogenic cells, stimulating fibrosis and mineralization of the ECM, subsequently closing a vicious cycle between vascular stiffness and vascular calcification. These two processes in combination represent important predictors of CV morbidity and mortality and initiate end-organ failure in multiple organs, including brain and kidneys [59,60]. In addition, hypertension is implicated in this interplay, as it promotes extracellular remodeling by accelerating type 1 collagen, fibronectin, and proteoglycan accumulation [61]. In fact, as discussed by McEniery et al., in comparison to normotensive patients, patients with isolated systolic hypertension present with increased calcification of both the abdominal and thoracic aorta [62]. Finally, a recent study, which was comprised of 10-year monitoring of ambulatory blood pressure in older hypertensives, revealed that 24 h pulse pressure better predicts mortality than 24 h systolic blood pressure; it is warranted that arterial stiffness reduction gains as much attention as lowering blood pressure in future clinical perspectives of CAD management [6].

## 3. MGP and Its Conformations

**Figure 1:**
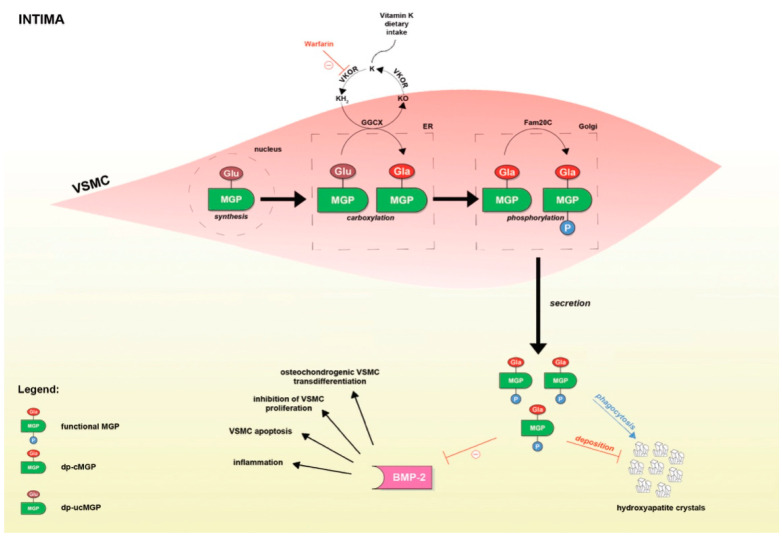
Post-translational modifications of MGP and the mechanisms by which MGP is implicated in vascular calcification. Red lines depict inhibition of the process, whereas the blue line indicate stimulation.

MGP, a small 12 kDa vitamin K-dependent protein, has been shown to play an important role in inhibition of both intimal and medial vascular calcification (Figure 1) [7]. It has been demonstrated that MGP is the most powerful natural inhibitor of calcification in the human body [52,53,63]. In fact, mice with knockout of the MGP gene die within 2 months as a result of widespread arterial calcification that leads to disintegration and rupture of the arterial wall [64]. In order to exert its functions, two post-translational modifications of MGP are warranted.

The first modification is a vitamin K-dependent γ-glutamate carboxylation at positions 2, 37, 41, 48, and 52 [65]. This modification enables binding of MGP to the crystal nuclei in hydroxyapatite and empowers MGP to binding and inhibition of the BMP-2, the aforementioned osteogenic growth factor that stimulates vascular calcification [66]. Binding of MGP to hydroxyapatite crystals abrogates their accumulation within the arterial wall and stimulates macrophages to promote phagocytosis and apoptosis of the newly formed MGP-hydroxyapatite complex [31]. On the other hand, MGP creates a complex with BMP-2 as well, thus preventing the binding of BMP-2 to its high-affinity receptor and preventing downstream signals that will lead to vascular calcification [67].

On the other hand, unlike carboxylation, phosphorylation of the serine residues at positions 3, 6, and 9 by a Golgi-casein kinase is a non-vitamin K-dependent process that seems to enable MGP to regulate the secretion of MGP into the extracellular environment [68]. In addition, since MGP retains its affinity for hydroxyapatite after thermal decarboxylation and ucMGP can also be seen at sites of calcification, it seems that these negatively charged carboxylated residues may also affect binding of MGP to calcium salts [69,70,71,72]. Of important note, apart from the phosphorylation state, plasma concentrations of MGP may also depend upon synthesis and degradation of MGP [65].

Based on the state of carboxylation and/or phosphorylation, various species of MGP may exist in circulation: phosphorylated-carboxylated MGP (p-cMGP), phosphorylated uncarboxylated MGP (p-ucMGP), dephosphorylated-carboxylated MGP (dp-cMGP), and dp-ucMGP. It is now obvious that their respective affinity for calcium salts and concomitant calcification-inhibitory activity may differ widely. This is of important relevance, as the levels of circulating MGP species may reflect the degree of calcification, or more accurately, inhibition of calcification in the vascular wall. Moreover, respecting the fact that carboxylation depends upon vitamin K, these may also reflect the availability of vitamin K present in the vascular wall.

Apart from the well-established role in vascular calcification inhibition, growing evidence suggests that activated MGP is implicated in preserving the structure and function of multiple organs, including the retina, bones and cartilages, kidney, and heart [73,74,75,76,77,78,79,80]. In line with this, the presence of inactive forms of MGP was observed in various pathologies. For instance, in our previous study, we found elevated dp-ucMGP levels in patients with inflammatory bowel disease (IBD), suggesting the involvement of MGP in IBD pathophysiology through inflammation process and disease activity [81]. In addition, Vilovic et al. demonstrated elevated dp-ucMGP levels in patients with obstructive sleep apnea (OSA), bringing further evidence to the complex interrelation between OSA and bone metabolism [82]. The widespread implications of MGP throughout the whole human body address the need for further clarification of MGP effects, especially with respect to consequences of its functional impairment.

## 4. MGP in Coronary Artery Disease (CAD)

Early reports suggested increased expression of MGP in human atherosclerotic lesions, paving a way for establishment of its role in this setting [83]. In apoE−/− mouse models, overexpression of functional MGP reduced both intimal and medial calcification of atherosclerotic plaques, whereas deletion of the MGP gene led to accelerated intimal calcification of plaque in the same mouse model [84]. In line with this, warfarin treatment of apoE−/− mice exhibited plaque calcification already after 1 week of administration, indicating that mechanisms that operate in developing plaques limiting pro-calcifying processes are vitamin K dependent [85]. As we discussed, vitamin K is necessary for post-transcriptional modification of MGP, and the above-noted processes seem to be mediated by MGP-induced BMP-2 suppression [86]. Moreover, in a study by van Gorp et al., the authors demonstrated that warfarin treatment significantly increased ucMGP in atherosclerotic lesions as compared to both control and dabigatran treatment in apoE−/− mice [87]. Additionally, it has been demonstrated in the same study that ucMGP significantly correlates with vascular calcification. However, Rattazzi et al. reported that warfarin, but not rivaroxaban, could induce calcific valve degeneration, yet that neither of the two significantly affects the progression of atherosclerosis in apoE−/− mice [88].

Given the widespread use of warfarin, a vitamin K antagonist (VKA) that affects the functionality of MGP, a doubt was raised with regard to the safety of this medication. In fact, Schurgers et al. investigated the effects of VKA treatment on the coronary calcium score in patients with suspected CAD who underwent multidetector computed tomography, demonstrating that both the use and duration of warfarin treatment correlate with coronary artery plaque calcification [85]. In line with this, Roijers et al. found a positive correlation between calcification of human coronary artery plaques and ucMGP expression in the plaque, demonstrating that mechanisms of warfarin-mediated accelerated plaque calcification are similar to that in the aforementioned mouse model [89]. Furthermore, Dalmeijer et al. established an association between CAC, as assessed by the Agatston score, and total ucMGP and dp-ucMGP, but not dp-cMGP, further substantiating the role of vitamin K in this setting [88]. In fact, multiple authors suggest that dp-ucMGP may serve as a biomarker of vascular vitamin K status with multiple clinical implications [65,90]. For instance, in our unpublished observations, we noticed that dp-ucMGP is associated with increased bleeding risk in patients with myocardial infarction (MI), suggesting a viable use of dp-ucMGP as an adjunctive biomarker complementary to the established bleeding scores.

Importantly, respecting that coronary artery calcium (CAC) scores improve CV risk discrimination, reclassifying a proportion of intermediate risk individuals, and that dp-ucMGP may reflect vascular calcification at a very early stage, Vassalle et al. argued that plasma dp-ucMGP levels could be used in CV risk assessment as an alternative to CAC [91,92]. Nevertheless, each novel biomarker should firstly be assessed depending on its appropriateness to answer several fundamental questions in order to evaluate its clinical relevance. Firstly, a biomarker must provide additional information beyond traditional biomarkers. Secondly, it has to be established to which group of patients should the marker be applied, and at which point in time should the biomarker be measured. Observations from our previous study even suggest the prognostic role of dp-ucMGP in CAD, as we demonstrated markedly higher dp-ucMGP plasma levels among NSTEMI patients at higher risk of in-hospital mortality, as assessed by the Global Registry of Acute Coronary Events (GRACE) score, an in-hospital mortality risk score holding a IIa recommendation in the current European Society of Cardiology (ESC) guidelines [93]. However, earlier studies reported rather conflicting data with respect to the association between elevated dp-ucMGP and poor outcomes in CV diseases. Mayer et al. demonstrated that the dp-ucMGP plasma levels were associated with all cause and CV mortality, with dp-ucMGP strongly predicting mortality in patients with lower CV risk [94]. Similar observations were also reported in populations with diabetes, and risk assessment with dp-ucMGP was independent of the classical risk factors and vitamin D status [95,96]. On the other hand, Dalmeijer et al. reported that the dp-ucMGP levels were not associated with increased CAD risk in their prospective case-cohort study [97]. Furthermore, a Mendelian randomization study conducted on the Flemish population showed that higher circulating dp-ucMGP predicts total, non-cancer, and CV mortality but lower coronary risk, remarking that non-cancer mortality and coronary events associations are likely causal [98]. In addition, the authors reported that the dp-ucMGP plasma levels in range between 1.4 and 4.6 μg/L are optimal in this setting, as they yield the lowest risk of mortality and macrovascular CVD.

In a study by Zwakenberg et al., the authors selected and analyzed 100 samples from the Athero-Express biobank in order to examine the interrelation of plasma MGP and plaque characteristics, as well as to compare plaque and plasma MGP [99]. The study showed that neither the dp-ucMGP nor total ucMGP plasma concentrations reflect the plaque ucMGP levels, and that the elevated dp-ucMGP levels are associated with less plaque hemorrhage, suggesting increased plaque stability. On the contrary, in the aforementioned study by Schurgers et al., the authors demonstrated that the use of VKAs enhances features of plaque instability by preventing post-translational modifications of MGP [85]. However, as BMP is implicated in the signaling networks regulating inflammation, SMC differentiation, and apoptosis, the authors concluded that VKAs could potentially affect the plaque phenotype on a more profound level, rather than solely accelerating the process of coronary plaque calcification [100,101,102,103,104]. According to these observations, one could infer that VKA use may be a risk factor for acute coronary events, which would be contrary to the available data, as the relatively safe profile of VKAs suggests differently. However, there is a possibility that the harmful effects of VKAs are masked by their inhibitory effects on the coagulant system, an important factor of atherothrombosis, addressing the need for alternative anticoagulants that do not interfere with the vitamin K cycle [105]. The role of MGP in the setting of CAD was also explored in our recent study [106]. For the first time, we demonstrated that patients with non-ST elevation MI (NSTEMI) have significantly higher circulating levels of dp-ucMGP then the ST elevation MI (STEMI) counterparts [106]. We hypothesized that higher circulating dp-ucMGP levels might reflect more calcified coronary lesions and a higher vascular calcification burden in patients with NSTEMI, indicating a difference in plaque pathobiology between STEMI and NSTEMI. Nonetheless, further large-scale studies are needed to substantiate these notions. Studies dealing with the role of MGP in the context of prognosis in patients with CAD are summarized in Table 2.

## 5. Conclusions

To summarize, although the role of MGP as a vascular calcification inhibitor has been well established, implications of this small protein and its various conformations in development of CAD still remains elusive. The main obstacle in defining the proper role of MGP in this setting is the dual role of calcification in atherosclerotic plaque development. Yet, it is clear that dp-ucMGP can reflect the vascular vitamin K status. Hence, dp-ucMGP might find its application as a biomarker of microvascular health and assist in clinical decision making with respect to the initiation of vitamin K supplementation. In addition, accumulating data, suggesting that VKAs could affect the plaque phenotype by interfering with the signaling networks regulating inflammation, SMC differentiation, and apoptosis, address the need for more vigilant prescription of these medications.

## Figures and Tables

**Table 1:** Notable inducers and inhibitors of vascular calcification.

Role	Molecule	Mechanism	Evidence
Vascular calcification inducers	BMP-2	Contributes to the transdifferentiation of VSMCs into osteochondrogenic cells; induces osteoblast differentiation; enhances apoptosis, oxidative stress and inflammation in VSMCs	[33,34,35]
	ALP	Its activity is important for hydroxyapatite formation	[36]
	FGF-23	Increases osteoblastic marker expression in VSMCs *	[37,38]
	Runx2	Transcription factor—increases expression of osteogenic genes	[39,40]
	PDK4	Induces osteogenic differentiation of VSMCs	[41,42]
	Cathepsin K	Degrades organic bone matrix in osteoclasts	[43,44]
Vascular calcification inhibitors	Osteoprotegerin	Interferes with RANK-RANKL interaction	[45,46]
	Osteopontin	Strong binding affinity for hydroxyapatite	[47,48]
	Fetuin-A	Binds to early calcium phosphate crystals inhibits growth and deposition	[49,50]
	BMP-7	Reduces transformation to VSMC osteogenic phenotypes	[51]
	MGP	Binding to hydroxyapatite; BMP-2 binding and inhibition	[52,53]

* Data regarding the role of FGF-23 in vascular calcification is conflicting. Abbreviations: BMP: bone morphogenetic protein; ALP: alkaline phosphatase; FGF-23: fibroblast growth factor-23; Runx2: runt-related transcription factor 2; PDK4: Pyruvate Dehydrogenase Kinase; VSMCs: vascular smooth muscle cells; MGP: Matrix Gla protein.

**Table 2:** Longitudinal studies relating the plasma MGP levels to cardiovascular disease and mortality.

Study	Study Population	Measured Outcomes (Median Duration)	Results
Dalmeijer et al. [93]	518 DM type II patients	HR_SD_ of dp-ucMGP, t-ucMGP and dp-cMGP for CVD, CHD, PAD, HF and stroke adjusted for sex, age, BMI, waist-to-hip ratio, blood pressure, total cholesterol, smoking, physical activity, and education (11.2 y)	Significant HR_SD_ ^1^: dp-ucMGP: 1.21 (1.06–1.38) for CVD; 1.33 (1.07–1.65) for PAD; 1.75 (1.42–2.17) for HF dp-cMGP: No significant HR_SD_ t-ucMGP: No significant HR_SD_
Dalmeijer et al. [95]	1154 incident cases of CHD and 380 of stroke + 1406 random participants (EPIC-NL)	HR_SD_ of dp-ucMGP for CHD risk and stroke (11.5 y)	HR_SD_ of dp-ucMGP: CHD: 1.00 (0.93–1.07) Stroke: 0.98 (0.90–1.08)
Keyzer et al. [107]	518 stable kidney transplant recipients	HR highest vs. lowest tertile of dp-ucMGP for TM and transplant failure (9.8 y)	HRs highest vs. lowest tertile of dp-ucMGP: TM: 3.10 (1.87–5.12) Transplant failure: 2.61 (1.22–5.57)
Liu et al. [96]	2318 FLEMENGHO participants	HR associated with dp-ucMGP doubling for TM, CVM, CVD and CHD adjusted for sex, age, body mass index, systolic blood pressure, heart rate, smoking and drinking, total cholesterol, DM, antihypertensive drug treatment, and history of CVD (14.1 y)	Significant HRs for dp-ucMGP doubling: TM: linear/squared term [1.06 (1.01–1.11)/1.02 (1.01–1.03)] CVM: 1.14 (1.01–1.28) CVD: No significant HRs CHD: No significant HRs
Mayer et al. [94]	799 patients with myocardial infarction, coronary revascularization or first ischemic stroke	HR highest vs. other quartiles of dp-ucMGP and dp-cMGP for TM and CVM (5.6 y)	HRs for highest quartile vs. Q1–Q3: dp-ucMGP: TM: 1.89 (1.32–2.72) CVM: 1.88 (1.22–2.90) dp-cMGP: TM: 1.76 (1.18–2.61) CVM: 1.79 (95% CI, 1.12–2.57)
Riphagen et al. [108]	4275 PREVEND participants	HR associated with dp-ucMGP doubling for TM and CVM, adjusted for ^2^ (8.5 y)	HRs for dp-ucMGP doubling: TM: linear/squared term [0.33 (0.17–0.66)/1.08 (1.03–1.13)] CVM: linear/squared term [0.17 (0.05–0.58)/1.11 (1.03–1.20)]
Schurgers et al. [109]	107 patients with CKD	RR of dp-ucMGP median (>921 p·mol/L) for TM adjusted for age, CKD stage or hemoglobin	RR for TM: 2.85 (1.36–5.90); significance lost in multivariable-adjusted models
Ueland et al. [110]	147 patients with symptomatic severe aortic stenosis	HR high versus low dp-cMGP and dp-ucMGP concentration for TM (23 months)	HRs high vs. low: dp-ucMGP: 9.33 (2.67–32.51) dp-cMGP: No significant HRs
Ueland et al. [111]	179 patients with chronic HF	HR_SD_ of dp-ucMGP for TM, fatal HF and heart transplant (2.9 y)	HR_SD_ of dp-ucMGP: TM: No significant HR_SD_ Fatal HF: 5.62 (2.05−15.5) Heart transplant: No significant HR_SD_

^1^ HR per SD. HRs and RRs are presented as HR (95% CI) ^2^ adjusted for ethnicity, sex, age, BMI, SBP, smoking, eGFR, total-to-HDL serum cholesterol ratio, CRP, albuminuria, use of antihypertensive drugs and warfarin, DM, history of CVD, and education.

## Data Availability

The data presented in this study are available on request from the corresponding author.

## Abbreviations

BMP	bone morphogenic protein
ALP	alkaline phosphatase
FGF-23	fibroblast growth factor-23
Runx2	RUNX family transcription factor 2
PDK4	pyruvate dehydrogenase kinase 4
MGP	matrix Gla protein
VSMC	vascular smooth muscle cell
BMP-2	bone morphogenetic protein-2
GGCX	gamma-glutamyl carboxylase
VKOR	vitamin K epoxide reductase
KH2	vitamin KH2
KO	vitamin KO
Fam20C	family with sequence similarity 20, member C
dp-cMGP	dephosphorylated-carboxylated MGP
dp-ucMGP	dephosphorylated-uncarboxylated MGP
ER	endoplasmic reticulum
HR	hazard ratio
RR	relative risk
CHD	coronary heart disease
CKD	chronic kidney disease
CVD	cardiovascular disease
CVM	cardiovascular mortality
DM	diabetes mellitus
eGFR	glomerular filtration rate estimated from serum creatinine
HF	heart failure
BMI	body mass index
